# Three-dimensional ultrastructural imaging reveals the nanoscale architecture of mammalian cells

**DOI:** 10.1107/S2052252517017912

**Published:** 2018-01-10

**Authors:** Shengkun Yao, Jiadong Fan, Zhiyun Chen, Yunbing Zong, Jianhua Zhang, Zhibin Sun, Lijuan Zhang, Renzhong Tai, Zhi Liu, Chunying Chen, Huaidong Jiang

**Affiliations:** a School of Physical Science and Technology, ShanghaiTech University, 393 Middle Huaxia Road, Pudong, Shanghai 201210, People’s Republic of China; b iHuman Institute, ShanghaiTech University, 393 Middle Huaxia Road, Pudong, Shanghai 201210, People’s Republic of China; c CAS Key Laboratory for Biomedical Effects of Nanomaterials and Nanosafety, National Center for Nanoscience and Technology of China, No. 11 ZhongGuanCun BeiYiTiao, Beijing 100190, People’s Republic of China; d State Key Laboratory of Crystal Materials, Shandong University, 27 Shanda Nanlu, Jinan, Shandong 250100, People’s Republic of China; e Shanghai Synchrotron Radiation Facility, Shanghai Institute of Applied Physics, Chinese Academy of Science, 239 Zhangheng Road, Pudong New District, Shanghai 201204, People’s Republic of China

**Keywords:** cellular imaging, X-ray tomography, equally sloped tomography, dual-energy contrast, computed tomography, nanoscience, X-ray microscopy, imaging, structure determination

## Abstract

Knowledge of the interactions between nanomaterials and large-size mammalian cells, including cellular uptake, intracellular localization and translocation, has greatly advanced nanomedicine and nanotoxicology. Imaging techniques that can locate nanomaterials within the structures of intact large-size cells at nanoscale resolution play crucial roles in acquiring this knowledge. Here, the quantitative imaging of intracellular nanomaterials in three dimensions was performed by combining dual-energy contrast X-ray microscopy and an iterative tomographic algorithm termed equally sloped tomography (EST). Macrophages with a size of ∼20 µm that had been exposed to the potential antitumour agent [Gd@C_82_(OH)_22_]_*n*_ were investigated. Large numbers of nanoparticles (NPs) aggregated within the cell and were mainly located in phagosomes. No NPs were observed in the nucleus. Imaging of the nanomedicine within whole cells advanced the understanding of the high-efficiency antitumour activity and the low toxicity of this agent. This imaging technique can be used to probe nanomaterials within intact large-size cells at nanometre resolution uniformly in three dimensions and may greatly benefit the fields of nanomedicine and nanotoxicology.

## Abstract 2

A single-cell imaging technique has been demonstrated by combining dual-energy contrast X-ray microscopy and an iterative tomographic algorithm called equally sloped tomography. The technique can simultaneously observe cellular ultrastructures and nanomaterial distributions.

## 1. Introduction   

Nanotechnology has dramatically increased the intentional and unintentional exposure of humans to nanomaterials, which generate various biological effects. Some nanomaterials exhibit diagnostic and therapeutic effects for certain diseases in medical applications, whereas others can cause possible adverse effects (Chauhan & Jain, 2013 ▸; Colvin, 2003 ▸; Oberdörster *et al.*, 2005 ▸). These diverse effects are determined by the interactions between nanomaterials and biological systems, particularly at the subcellular level, including cellular uptake, intracellular localization and translocation (Cheng *et al.*, 2013 ▸; Chou *et al.*, 2011 ▸; Iversen *et al.*, 2011 ▸). Imaging techniques that can visualize nanomaterials within whole cells at nanometre resolution now play a crucial role in nano­medicine and nanotoxicology.

Electron microscopy has become the most commonly used technique for cellular studies to date because of its excellent resolving power. However, the tedious sample-preparation and mechanical slicing procedures make the nondestructive imaging of intact cells challenging (Medalia *et al.*, 2002 ▸; McIntosh *et al.*, 2005 ▸; Leis *et al.*, 2009 ▸). Optical microscopy facilitates the imaging of cells in a more rapid and simple manner, can be used to obtain videos of living cells and can achieve exquisite detail and molecular specificity *via* the use of fluorescent labelling technologies. However, the complete cellular context cannot be elucidated (Stephens & Allan, 2003 ▸; Dean & Palmer, 2014 ▸; Ntziachristos, 2010 ▸; Jia *et al.*, 2014 ▸; Huang *et al.*, 2009 ▸; Hell, 2003 ▸; Betzig *et al.*, 2006 ▸). Thus, important gaps exist between optical and electron microscopic techniques in terms of their spatial resolution, sample thickness, labelling requirements, contrast mechanism and quantitative capability. Several X-ray imaging approaches have made important contributions towards bridging this gap, including zone plate-based and lens-less X-ray microscopy (Kirz *et al.*, 2009 ▸; Larabell & Nugent, 2010 ▸; Le Gros *et al.*, 2005 ▸; Leis *et al.*, 2009 ▸; Pérez-Berná *et al.*, 2016 ▸; Conesa *et al.*, 2016 ▸; Varsano *et al.*, 2016 ▸). Dual-energy X-ray microscopy is a promising choice to explore the interactions between nanomaterials and cells, and can achieve quantitative nondestructive three-dimensional imaging of both cellular structures and nanomaterials at nanometre resolution simultaneously.

**Figure 1:**
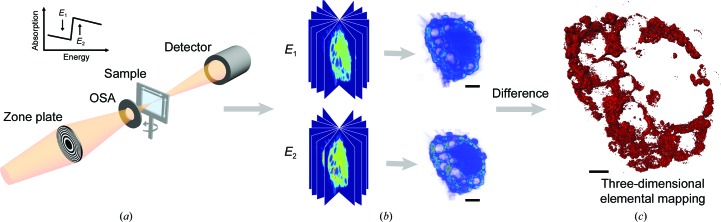
Schematic layout of the dual-energy imaging technique. (*a*) Two sets of projections were acquired by STXM at energies below and above the absorption edge of a specific element, (*b*) the projections were reconstructed by the EST algorithm separately (scale bar 4.0 µm) and (*c*) the quantitative three-dimensional distribution of the specific element was calculated based on the abrupt change in the absorption (scale bar 2.0 µm).

Dual-energy contrast X-ray microscopy is based on the abrupt change in the absorption when the energy of the incident X-rays changes from just below the absorption edge of a specific element to above it (Fig. 1 ▸). The observed differences between images collected at the two energies reflect the locations of element-specific nanomaterials (Zhang *et al.*, 2010 ▸; Rarback *et al.*, 1987 ▸). This method has been widely applied to many fields to investigate biological materials (Wang *et al.*, 2015 ▸; Conesa *et al.*, 2016 ▸; Chen *et al.*, 2014 ▸), organic materials (Johansson *et al.*, 2007 ▸; Hitchcock *et al.*, 2008 ▸), energy materials (Nelson *et al.*, 2011 ▸; Liu *et al.*, 2014 ▸; Kao *et al.*, 2013 ▸; Andrews & Weckhuysen, 2013 ▸) and clinical medical applications (Houk *et al.*, 1979 ▸; Lewis, 1997 ▸). This method can simultaneously image the ultrastructures of cells and intracellular nanomaterials, and thus can be used to investigate the interactions between nanomaterials and biological systems. However, its applications for cellular imaging are limited by the sensitivity of biological specimens to irradiation and the robustness of the tomographic algorithms. In particular, the nanometre-resolution imaging of large-size mammalian cells is quite difficult because of sample preparation, the field of view of image techniques and the missing wedge problem in tomographic reconstruction.

We applied dual-energy contrast X-ray microscopy to explore the intracellular localization of nanomaterials in whole large-size mammalian cells. By combining scanning transmission X-ray microscopy (STXM) and a Fourier-based iterative tomographic algorithm termed equally sloped tomography (EST), we investigated the quantitative three-dimensional subcellular distribution of Gd@C_82_(OH)_22_ within a macrophage. Gd@C_82_(OH)_22_ is a promising antitumour agent that exhibits high-efficiency antitumour activities and low toxicity *in vitro* and *in vivo* (Meng, Wang *et al.*, 2010 ▸; Meng *et al.*, 2013 ▸; Li *et al.*, 2012 ▸; Chen *et al.*, 2005 ▸). The exact antitumour mechanism is not clear, although the antitumour activity is known to result from tumour microenvironment regulation instead of direct cytotoxicity to tumour cells (Yin *et al.*, 2008 ▸, 2009 ▸; Wang *et al.*, 2006 ▸; Meng, Xing *et al.*, 2010 ▸; Meng *et al.*, 2012 ▸; Liu *et al.*, 2009 ▸; Liang *et al.*, 2010 ▸). Macrophages play key roles in the microenvironment because of their diverse functions. They are crucial components of the innate immune system and are engaged in host defence activities, including the removal of dead cells and pathogens by phagocytosis, the secretion of cytokines and other factors, and the modulation of adaptive immunity by presenting antigens to lymphocytes (Weissleder *et al.*, 2014 ▸). Meanwhile, tumour-associated macrophages exert stimulating effects on tumour growth by establishing tumour stroma conditions (Ruffell *et al.*, 2012 ▸; Qian & Pollard, 2010 ▸). Thus, by investigating the subcellular distribution of this nanomedicine, we hope to improve our understanding of the antitumour activities of this agent.

## 2. Experimental   

### 2.1. Sample preparation   

For the *in vitro* experiments, mouse peritoneal macrophages were incubated and treated with Gd@C_82_(OH)_22_ nanoparticles (NPs; 50 µ*M*) on 100 nm carbon membranes for 3 h. The cells were fixed in cold 70% ethanol for 40 min and then gradually dehydrated in 85% ethanol for 15 min, 95% ethanol for 10 min and 100% ethanol for 10 min (Chen *et al.*, 2014 ▸). Well isolated macrophages were then selected by light microscopy and used for experiments.

### 2.2. Data acquisition   

The experiment was mainly performed on the Soft X-ray Spectromicroscopy Beamline (BL08U1A) at the Shanghai Synchrotron Radiation Facility (SSRF; Zhang *et al.*, 2015 ▸). The schematic layout of the STXM is presented in Fig. 1 ▸(*a*). Monochromatic X-rays were focused with a Fresnel zone plate (FZP) with an outer zone width of 30 nm. After an order-sorting aperture (OSA), a 50 nm focal spot was achieved. The X-ray transmission of the sample was then detected using a photomultiplier tube. A two-dimensional image was formed after a raster scan of the full sample.

The macrophages grown on the carbon membrane were scanned through the focal plane perpendicular to the beam direction with a 50 nm step size and a 5 ms dwell time. After one projection had been obtained, the sample stage was rotated. For tomographic reconstruction, projections were measured at 46 equally sloped angles over a range of ±79.4°. The missing wedge caused by the interference of the stage holder with the incident X-rays was limited to 20°. Equally sloped angles were chosen so that EST could be used. The energies were determined based on the total electron yield (TEY) signals (*i.e.* 1189 and 1186 eV; Supplementary Fig. S2). Two data sets were measured above and below the Gd *M*_2_ edge (Supplementary Figs. S3 and S4). The sample was re-focused after the stage had been rotated and the energy changed. The total radiation dose delivered to the macrophage was minimized to ∼4.43 × 10^6^ Gy, and little structural change was observed during the experiment. To assess the effect of radiation damage, two projections were taken at 0° before and after full data sets had been acquired. The two projections were in good agreement, indicating that there was no detectable radiation damage to the macrophage (Supplementary Fig. S5).

XRF mapping of the same macrophage was performed on the Hard X-ray Micro-Focus Beamline (BL15U) at SSRF (Qiu *et al.*, 2011 ▸). A focal spot with a size of 200 nm and an energy of 8 keV was raster-scanned through the macrophage using a 200 nm step size, and the two-dimensional distribution of gadolinium (Gd) was determined.

### 2.3. Tomographic reconstruction   

EST is an iterative method that is based on the pseudo-polar fast Fourier transform (PPFFT; Averbuch, Coifman, Donoho, Israeli & Shkolnisky, 2008 ▸; Averbuch, Coifman, Donoho, Israeli, Shkolnisky *et al.*, 2008 ▸) and the oversampling method (Miao *et al.*, 2005 ▸; Jiang *et al.*, 2010 ▸). Instead of acquiring projections with constant angular increments, as in conventional tomography, projections obtained using the EST method are acquired using equally sloped increments. With PPFFT, projections with equally sloped increments are utilized to make mathematically exact three-dimensional reconstructions. When a limited number of projections are obtained using iteration between real space and Fourier space, reconstructions that are more precise than those produced by conventional tomography can be generated (Yao *et al.*, 2016 ▸). In real space, the negative density and density outside support are pushed to zero. In reciprocal space, Fourier coefficients with both magnitudes and phases, which are obtained from reconstructed projections, are updated at each iteration, whereas the other Fourier coefficients remain unchanged. The iteration is monitored by an error metric defined as the difference between the calculated Fourier coefficients and those obtained experimentally, and the algorithm is terminated when a maximum number of iterations is reached.

Before tomographic reconstruction, the projections were aligned to a common tilt axis. The tilt axes had an arbitrary shift along the *x* and *y* axes in the experiment, where *y* is the tilt axis and *z* is the beam direction. The shift was corrected using the centre-of-mass method, which has been tested experimentally. The aligned projections were then reconstructed using the EST method (Miao *et al.*, 2005 ▸; Scott *et al.*, 2012 ▸; Zhao *et al.*, 2012 ▸; Jiang *et al.*, 2010 ▸). In our reconstructions, we terminated the algorithm after 300 iterations, when the error function stabilized. The tomographic quality was verified by comparing the experimental and calculated projections (Supplementary Fig. S6), and three-dimensional structure information was collected at ∼75–80 nm resolution (Supplementary Figs. S7 and S8).

### 2.4. Dual-energy contrast X-ray microscopy   

When the energy of the incident X-rays changes from just below the absorption edge of a specific element to above it, the attenuation of the X-rays varies dramatically. This abrupt change is used in the dual-energy contrast-imaging method to obtain a quantitative distribution of the specific element. In this method, two images are acquired at the same tilt angles at *E*_1_ and *E*_2_, where *E*_1_ and *E*_2_ represent the energies below and above the absorption edge of a specific element. Differences between the two images contribute to confirming the presence of specific element, and the absorption of other elements can be neglected because the change in the photon energy is small.

(i) TEY, which detects the electric current caused by secondary electrons, was used to measure the X-ray absorption edge spectra of gadolinium (Gd), and the energies just below and above the absorption edge were determined.
(ii) Tomographic data acquisition was performed by STXM at the two selected energies.
(iii) The data sets were reconstructed separately with the EST algorithm.
(iv) Mapping of the intracellular nanomaterials was achieved by calculating the difference in the reconstructed three-dimensional volume after calibrating the position.
(v) The threshold was determined based on the intensity histogram (Supplementary Fig. S9) and was verified by the two-dimensional distribution of Gd obtained based on a two-dimensional projection from the calculated three-dimensional Gd distribution (Supplementary Fig. S10).

### 2.5. Organelle segmentation and three-dimensional volume rendering   

The reconstructed three-dimensional images were segmented manually and visualized using the *Amira* software package. The organelles in a cell have characteristic linear absorption coefficients because of the differences in their chemical compositions (McDermott *et al.*, 2012 ▸; Le Gros *et al.*, 2005 ▸; Larabell & Nugent, 2010 ▸). This property was used to segment three-dimensional images into subvolume regions that had similar linear absorption coefficients. The segmented regions were then identified based on the organelle morphologies, which were evaluated by other methods, such as light and electron microscopy techniques. To study the transport of NPs in the vacuoles, we focused on the segmentation of different types of vacuoles. Three types of vacuoles with specific densities and sizes were segmented and identified.

## 3. Results and discussion   

### 3.1. Three-dimensional ultrastructure of the macrophage   

**Figure 2:**
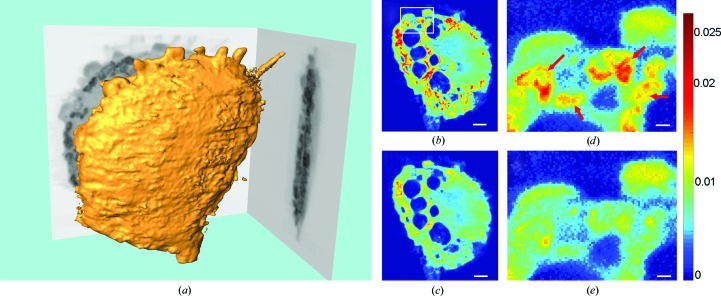
Three-dimensional structures of the macrophage. (*a*) The isosurface rendering at 1189 eV showing characteristic features of the macrophage, such as pseudopods, rough surfaces and flat shapes. These features indicate that the immune cell was in a highly active state. The same slices at 1189 eV (*b*) and 1186 eV (*c*) perpendicular to the beam direction reveal the subcellular structures, such as low-density vacuoles. Enlarged regions of the rectangle at 1189 eV (*d*) and 1186 eV (*e*) show differences in absorption because of the presence of [Gd@C_82_(OH)_22_]_*n*_. The scale bar in (*b*) and (*c*) is 2.0 µm and that in (*d*) and (*e*) is 0.5 µm.

A well isolated macrophage that had been treated with Gd@C_82_(OH)_22_ and grown on a carbon membrane was selected using a light microscope (Supplementary Fig. S1). The three-dimensional structure of the cell was obtained by combining dual-energy STXM and the EST algorithm. The tomographic images show that the macrophage, which had overall dimensions of ∼18.1 × 15.5 × 3 µm, exhibited distinctive features such as an irregular shape, a rough surface and branching pseudopods (Fig. 2 ▸ *a*), which coincide with the optical image.

In addition to the morphological analysis, which had higher resolution than the optical imaging, the internal structures of the macrophage were visualized by virtual sectioning into 50 nm thick slices. The colour in the slices represents different linear absorption coefficients: red corresponds to high values, yellow to medium values and blue to low values. Figs. 2 ▸(*b*) and 2 ▸(*c*) present two slices perpendicular to the beam direction at the two energies (*i.e.* 1189 and 1186 eV), and vacuoles with low linear absorption coefficients can clearly be observed. These vacuoles were distributed in the cytoplasm and had volumes of ∼1.7–6.3 µm^3^. Moreover, large numbers of dense round particles (*i.e.* primary lysosomes) with high linear absorption coefficients were evident, and their diameters were in the range ∼200–400 nm. The characteristic structures agree with previous results obtained *via* optical and electron microscopy (Papadimitriou & Ashman, 1989 ▸), indicating that the macrophage is in the active state. The state of the macrophage was further confirmed by the ability of Gd@C_82_(OH)_22_ to induce primary mouse macrophages to produce significant numbers of pro-inflammatory cytokines (Supplementary Table S1). The intracellular distribution of [Gd@C_82_(OH)_22_]_*n*_ could be distinguished qualitatively according to the differences in the linear absorption coefficient between the two slices (Figs. 2 ▸ *d* and 2 ▸ *e*, arrows). To quantitatively analyze the three-dimensional distributions of the nanomaterials, calculations based on the abrupt change in the absorption at the two energies were performed.

### 3.2. Three-dimensional intracellular distribution of [Gd@C_82_(OH)_22_]_*n*_   

**Figure 3:**
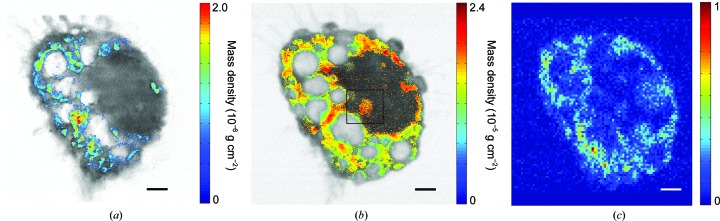
Distribution of [Gd@C_82_(OH)_22_]_*n*_ in the macrophage. (*a*) Quantitative distribution of [Gd@C_82_(OH)_22_]_*n*_ in a 50 nm thick slice. (*b*) Distribution of [Gd@C_82_(OH)_22_]_*n*_ in a projection perpendicular to the beam direction. (*c*) XRF of the same cell mapping Gd. The exact three-dimensional location of [Gd@C_82_(OH)_22_]_*n*_ can be determined in (*a*). Furthermore, the high spatial resolution in (*a*) avoids the ambiguity in (*c*), which is caused by the low spatial resolution and self-absorption of fluorescence signals (scale bar 2.0 µm).

**Figure 4:**
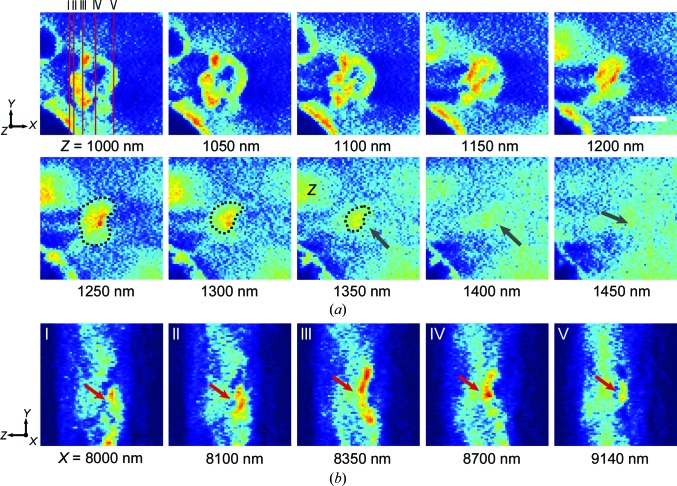
Positional determination of nanomaterials in the nuclear region. The exact three-dimensional distribution of [Gd@C_82_(OH)_22_]_*n*_ in the nuclear region distinguished by sectioning in two orthogonal directions, where the *z* direction is the beam direction. (*a*) Successive slices along the *z* direction. The change in the linear absorption coefficient was used to determine the exact position of the nanomaterials, and lysosomes containing nanoparticles stick together and remain on the surface of the nucleus. The interfaces of the nucleus and vacuoles with nanomaterials are shown by black arrows. (*b*) Slices along the *x* direction at five positions (I, II, III, IV and V), as shown in (*a*). In I, II and V cracks are obvious (red arrows), indicating that the nanomaterials are located on the surface of the nucleus (scale bar 1.0 µm).

The three-dimensional intracellular distribution of [Gd@C_82_(OH)_22_]_*n*_ was determined and virtually quantitated slice by slice. Fig. 3 ▸(*a*) displays a 50 nm thick slice in which [Gd@C_82_(OH)_22_]_*n*_ is aggregated in the macrophages and exhibits a characteristic distribution. Compared with the two-dimensional projected distribution (Fig. 3 ▸ *b*), the problems with ambiguity resulting from the overlap of structures along the beam direction were solved. To verify the exact locations of the nanoparticles (NPs) in the nuclear regions, the rectangular region in Fig. 3 ▸(*b*) was analyzed, slice by slice, in two perpendicular directions (Fig. 4 ▸). A cluster of NPs was found on the surface of the nucleus and was distributed into one large and four small vacuoles, in which the density and distribution of [Gd@C_82_(OH)_22_]_*n*_ were different. This macrophage was also subjected to hard X-ray fluorescence (XRF) microscopy to compare its effectiveness with that of dual-energy contrast microscopy (Fig. 3 ▸ *c*). Although the general distribution of [Gd@C_82_(OH)_22_]_*n*_ could be approximated, the distribution was indistinguishable in some regions, especially near the nucleus, because of the limited resolution and self-absorption of the XRF signals.

### 3.3. Quantitative observation of the ultrastructures of the macrophage and nanomaterial distributions simultaneously   

**Figure 5:**
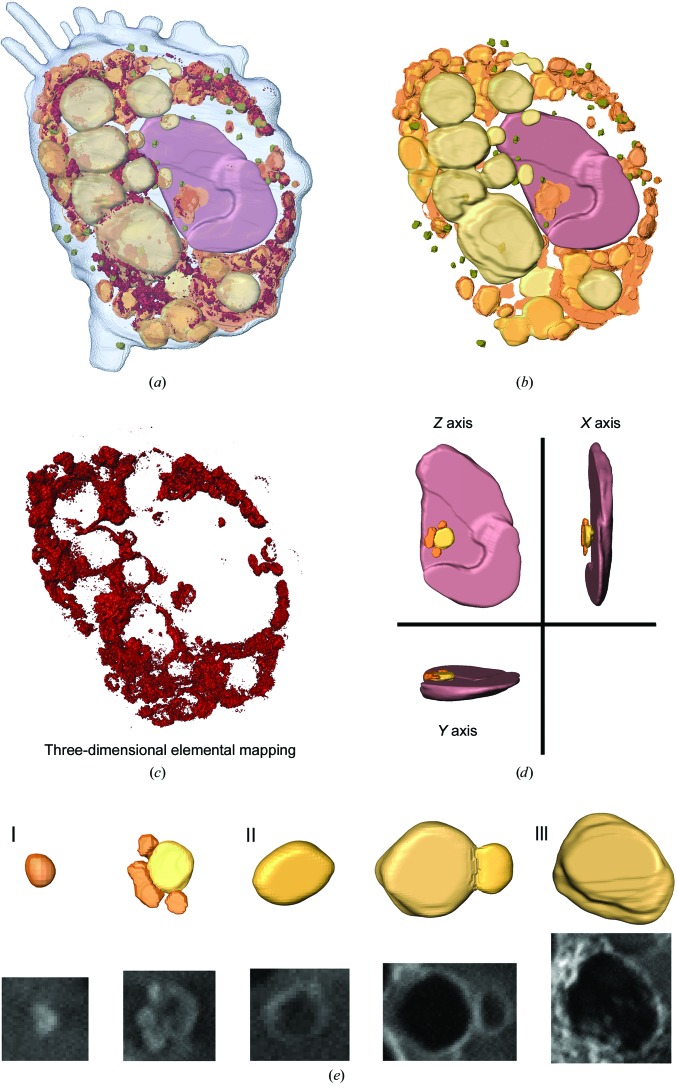
The intracellular distribution of nanomaterials. (*a*) Volume rendering of the reconstructed macrophage showing [Gd@C_82_(OH)_22_]_*n*_ (dark red), nucleus (brown) and different types of lysosomes (yellow). (*b*) Organelle segmentation based on differences in the linear absorption and specific morphology. (*c*) Three-dimensional distribution of [Gd@C_82_(OH)_22_]_*n*_, showing that the NPs aggregate and exhibit a characteristic distribution in the cell. (*d*) Enlarged view of the nucleus in three dimensions showing that the NPs were distributed in four separated lysosomes on the surface of the nucleus. (*e*) Three different types of lysosomes were distinguished based on the density and distribution of [Gd@C_82_(OH)_22_]_*n*_, which may reflect the intracellular transportation of the nanomaterials.

Three-dimensional images of the macrophage were segmented based on differences in the linear absorption coefficient and specific morphology (Figs. 5 ▸ *a* and 5 ▸ *b*). [Gd@C_82_(OH)_22_]_*n*_ was taken up effectively by the macrophage and redistributed at the subcellular level. Fig. 5 ▸(*c*) presents the distribution of [Gd@C_82_(OH)_22_]_*n*_ in the cell. Large numbers of aggregated NPs were distributed in the cytoplasm. The total mass of NPs was ∼1.2 × 10^−10^ g, and the volume ratio of NPs to the macrophage was ∼29%. The nanomaterials were distributed only in phagocytic vesicles, and no NPs were observed in other organelles, including the nucleus. Phagocytic vesicles have different volumes and densities (Fig. 5 ▸ *b*), and the distributions of the nanomaterials in these vesicles also varied (Fig. 5 ▸ *e*). Small vesicles tended to stick together and become larger ones. As the volume size increased, the density of the vesicles decreased and NPs distributed around the membrane. [Gd@C_82_(OH)_22_]_*n*_ was exclusively observed within cytoplasmic vesicles. Highly agglomerated particles were primarily located in the vesicle periphery and formed ring-shaped structures. The redistribution of NPs into different vesicles and the changes in the volume size and density of phagocytic vesicles may imply the rearrangement and fusion of vesicles and NPs in the vesicles of macrophages at the subcellular level.

Gd@C_82_(OH)_22_, an endohedral hydroxylated metallo­fullerene, is a promising antitumour agent that has highly efficient antitumour activities and low toxicity both *in vitro* and *in vivo* (Chen *et al.*, 2005 ▸; Meng *et al.*, 2013 ▸; Meng, Wang *et al.*, 2010 ▸; Li *et al.*, 2012 ▸). Previous studies mainly focused on the interaction between the NPs and individual organisms as well as the distribution of the NPs in different organs (Meng *et al.*, 2012 ▸, 2013 ▸; Li *et al.*, 2012 ▸; Zhang *et al.*, 2011 ▸; Meng, Wang *et al.*, 2010 ▸; Liang *et al.*, 2010 ▸; Yin *et al.*, 2008 ▸, 2009 ▸; Liu *et al.*, 2009 ▸; Wang *et al.*, 2006 ▸; Chen *et al.*, 2005 ▸). The interaction between NPs and cells at the subcellular level is substantially more important for understanding the antitumour mechanism. To better understand the antitumour mechanisms of [Gd@C_82_(OH)_22_]_*n*_ on the subcellular scale, the quantitative three-dimensional distribution of the NPs in macrophages was investigated by combining dual-energy STXM and the EST algorithm. Here, characteristic structures were observed, such as pseudopods and large vacuoles, thus indicating that [Gd@C_82_(OH)_22_]_*n*_ may be an immune potentiator that initiates the immune activities of macrophages. Moreover, the distinctive properties of the NPs facilitate their uptake by macrophages. Gd@C_82_(OH)_22_ is water-soluble and tends to aggregate into clusters because of the modification of the hydroxyl groups on the fullerene cage (Meng *et al.*, 2013 ▸). Their biocompatibility is also improved as the outer surface of the NPs is usually surrounded by water molecules because of hydrogen bonding. The specific structures and surface properties of the NPs and the immune stimulation of the macrophage may contribute to the high efficiency for cell uptake. Large amount of nanoparticles are easily incorporated into the cell, which suggest high efficiency of the nanomedicine. Additionally, the low toxicity of [Gd@C_82_(OH)_22_]_*n*_ relative to conventional chemotherapy also makes this species a promising biomedical candidate. The toxicity of NPs is closely related to their subcellular distribution. [Gd@C_82_(OH)_22_]_*n*_ was taken up by the macrophage and mainly located in phagosomes, not in the nucleus. This specific distribution in organ­elles that are relatively less important for the activities of the cell, to some extent, suggest low toxicity of the potential nanomedicine.

Dual-energy contrast X-ray microscopy shows obvious advantages for investigating intracellular nanomaterials. Firstly, its relatively high three-dimensional uniform resolution enables studies of large-size cells at the subcellular level. As shown in Fig. 3 ▸, the subcellular distribution of [Gd@C_82_(OH)_22_]_*n*_ was clearly revealed by dual-energy contrast STXM, whereas that determined by XRF remained ambiguous. Although scanning XRF microprobes have been used to generate two-dimensional projected elemental distributions with resolutions below 60 nm (Zhao *et al.*, 2014 ▸; de Jonge & Vogt, 2010 ▸), the three-dimensional resolution is limited to ∼400 nm (de Jonge *et al.*, 2010 ▸). The limited resolution of XRF makes investigating the subcellular distribution of trace elements difficult. In our experiment, the ultimate resolution of the three-dimensional STXM reconstruction was limited by the size of the focal spot. By minimizing the spot size, a much higher resolution can be achieved. Moreover, the missing wedge problem was eliminated by using an iterative tomographic algorithm (Supplementary Fig. S7). Secondly, obtaining the three-dimensional distribution of [Gd@C_82_(OH)_22_]_*n*_ was much easier. Although three-dimensional elemental mapping could be achieved by combining XRF and tomography, acquiring data and correcting for the self-absorption were difficult. Thirdly, the radiation damage resulting from the experiment was limited by the combination of STXM and EST. The total time required for data acquisition was the primary limitation of this method. However, this time was mainly composed of user actions because of the manual nature of this prototype setup. Indeed, a substantial amount of time was wasted on re-focusing the setup when the energy and angle were changed. The time required could be significantly decreased by developing an automated focusing system. X-rays are suited to investigating trace elements in whole biological systems, such as unsectioned cells (Uchida *et al.*, 2009 ▸; Schneider *et al.*, 2010 ▸), because of the higher penetration of X-rays compared with electrons or visible light sources. Combining X-ray microscopy and spectroscopy makes the imaging of intracellular nanomaterials possible with high spatial and chemical resolution, which would greatly benefit the field of nanoscience.

In this study, we demonstrated the quantitative imaging of intracellular nanomaterials in three dimensions on the nanoscale by dual-energy contrast X-ray tomography. The intracellular distribution of nanomaterials was further confirmed by using X-ray fluorescence microscopy. This work shows the advantages of the multiple-model approach, indicating the trend and the future of X-ray imaging. By using the multiple-model approach, each modality contributes unique information on the specimen. The complementary data are combined and consequently yield a more complete picture of the specimen. Here, STXM was used to image cells and intra­cellular nanomaterials. In this modality, image contrast is generated by the differential attenuation of specimen illumination by the cell contents. The dual-energy scan can provide elemental specificity. In addition, the phase-contrast mode in a standard STXM setup has recently been developed in different ways, such as through the use of a pixelated detector (Liu *et al.*, 2013 ▸). The phase-contrast mode will take advantage of the shift in X-ray phase on transmission of the sample and be helpful for visualizing the subcellular structures and achieving high-quality imaging. Furthermore, with the development of synchrotron-radiation facilities, optical components and imaging methodologies, scanning hard X-ray fluorescence microscopy is gradually solving limitations such as the time-consuming data acquisition and low spatial resolution, showing the advantages in mapping multi-elemental content distributions in single cells down to trace amounts (Smith *et al.*, 2014 ▸; Liu *et al.*, 2016 ▸; Deng *et al.*, 2015 ▸). Therefore, the multiple-model approach combining different techniques will provide quantitative and qualitative information on the density distribution and ele­mental contents of interest in the context of the ultrastructures of samples.

## 4. Conclusions   

In summary, in this work quantitative three-dimensional intracellular imaging of nano­materials has been demonstrated. [Gd@C_82_(OH)_22_]_*n*_ was highly taken up by macrophages and was mainly distributed in lysosomes. The subcellular distribution of this nanomedicine advanced our understanding of the corresponding antitumour activities. The results confirmed the utility of dual-energy contrast X-ray microscopy for studying the interactions between nanomaterials and large-size mammalian cells. The advantages of this technique include the high-resolution three-dimensional subcellular location of nano­materials within intact cells and simple sample-preparation procedures without the need for labelling. Moreover, element-specific nanomaterials could be distinguished when images were acquired at the corresponding absorption edge. Although the resolution was limited to ∼75–80 nm in our experiment, nanometre resolution could be achieved with the development of light sources and optical elements. Dual-energy contrast X-ray microscopy could simultaneously reveal both the structures of cells and the subcellular distributions of nanomaterials at nanometre resolution. We anticipate that this method will have broad applications in nanomedicine and nanotoxicology.

## Supplementary Material

Supplementary Figures and Table.. DOI: 10.1107/S2052252517017912/mf5022sup1.pdf

## References

[bb1] Andrews, J. C. & Weckhuysen, B. M. (2013). *Chemphyschem*, **14**, 3655–3666. 10.1002/cphc.201300529 24038941

[bb2] Averbuch, A., Coifman, R. R., Donoho, D. L., Israeli, M. & Shkolnisky, Y. (2008). *SIAM J. Sci. Comput.* **30**, 764–784.

[bb3] Averbuch, A., Coifman, R. R., Donoho, D. L., Israeli, M., Shkolnisky, Y. & Sedelnikov, I. (2008). *SIAM J. Sci. Comput.* **30**, 785–803.

[bb4] Betzig, E., Patterson, G. H., Sougrat, R., Lindwasser, O. W., Olenych, S., Bonifacino, J. S., Davidson, M. W., Lippincott-Schwartz, J. & Hess, H. F. (2006). *Science*, **313**, 1642–1645. 10.1126/science.1127344 16902090

[bb5] Chauhan, V. P. & Jain, R. K. (2013). *Nat. Mater.* **12**, 958–962. 10.1038/nmat3792 PMC4120281 24150413

[bb7] Chen, C. *et al.* (2005). *Nano Lett.* **5**, 2050–2057. 10.1021/nl051624b 16218736

[bb6] Chen, Z. Y., Liu, Y., Sun, B. Y., Li, H., Dong, J. Q., Zhang, L. J., Wang, L. M., Wang, P., Zhao, Y. L. & Chen, C. Y. (2014). *Small*, **10**, 2362–2372. 10.1002/smll.201302825 24619705

[bb8] Cheng, L.-C., Jiang, X., Wang, J., Chen, C. & Liu, R.-S. (2013). *Nanoscale*, **5**, 3547–3569. 10.1039/c3nr34276j 23532468

[bb9] Chou, L. Y., Ming, K. & Chan, W. C. (2011). *Chem. Soc. Rev.* **40**, 233–245. 10.1039/c0cs00003e 20886124

[bb10] Colvin, V. L. (2003). *Nat. Biotechnol.* **21**, 1166–1170. 10.1038/nbt875 14520401

[bb11] Conesa, J. J., Otón, J., Chiappi, M., Carazo, J. M., Pereiro, E., Chichón, F. J. & Carrascosa, J. L. (2016). *Sci. Rep.* **6**, 22354. 10.1038/srep22354 PMC4785355 26960695

[bb12] Dean, K. M. & Palmer, A. E. (2014). *Nat. Chem. Biol.* **10**, 512–523. 10.1038/nchembio.1556 PMC4248787 24937069

[bb13] Deng, J., Vine, D. J., Chen, S., Nashed, Y. S., Jin, Q., Phillips, N. W., Peterka, T., Ross, R., Vogt, S. & Jacobsen, C. J. (2015). *Proc. Natl Acad. Sci. USA*, **112**, 2314–2319. 10.1073/pnas.1413003112 PMC4345580 25675478

[bb14] Hell, S. W. (2003). *Nat. Biotechnol.* **21**, 1347–1355. 10.1038/nbt895 14595362

[bb15] Hitchcock, A. P., Johansson, G. A., Mitchell, G. E., Keefe, M. H. & Tyliszcak, T. (2008). *Appl. Phys. A*, **92**, 447–452.

[bb16] Houk, T. L., Kruger, R. A., Mistretta, C. A., Riederer, S. J., Shaw, C. G., Lancaster, J. C. & Flemming, D. C. (1979). *Invest. Radiol.* **14**, 270–278. 10.1097/00004424-197907000-00002 385547

[bb17] Huang, B., Bates, M. & Zhuang, X. (2009). *Annu. Rev. Biochem.* **78**, 993–1016. 10.1146/annurev.biochem.77.061906.092014 PMC2835776 19489737

[bb18] Iversen, T. G., Skotland, T. & Sandvig, K. (2011). *Nano Today*, **6**, 176–185.

[bb19] Jia, S., Vaughan, J. C. & Zhuang, X. (2014). *Nat. Photonics*, **8**, 302–306. 10.1038/nphoton.2014.13 PMC4224117 25383090

[bb20] Jiang, H., Song, C., Chen, C.-C., Xu, R., Raines, K. S., Fahimian, B. P., Lu, C.-H., Lee, T.-K., Nakashima, A., Urano, J., Ishikawa, T., Tamanoi, F. & Miao, J. (2010). *Proc. Natl Acad. Sci. USA*, **107**, 11234–11239. 10.1073/pnas.1000156107 PMC2895086 20534442

[bb21] Johansson, G. A., Tyliszczak, T., Mitchell, G. E., Keefe, M. H. & Hitchcock, A. P. (2007). *J. Synchrotron Rad.* **14**, 395–402. 10.1107/S0909049507029962 17717380

[bb22] Jonge, M. D. de, Holzner, C., Baines, S. B., Twining, B. S., Ignatyev, K., Diaz, J., Howard, D. L., Legnini, D., Miceli, A., McNulty, I., Jacobsen, C. J. & Vogt, S. (2010). *Proc. Natl Acad. Sci. USA*, **107**, 15676–15680. 10.1073/pnas.1001469107 PMC2936603 20720164

[bb23] Jonge, M. D. de & Vogt, S. (2010). *Curr. Opin. Struct. Biol.* **20**, 606–614. 10.1016/j.sbi.2010.09.002 20934872

[bb24] Kao, T. L., Shi, C. Y., Wang, J., Mao, W. L., Liu, Y. & Yang, W. (2013). *Microsc. Res. Tech.* **76**, 1112–1117. 10.1002/jemt.22273 23922210

[bb25] Kirz, J., Jacobsen, C. & Howells, M. (2009). *Q. Rev. Biophys.* **28**, 33. 10.1017/s0033583500003139 7676009

[bb26] Larabell, C. A. & Nugent, K. A. (2010). *Curr. Opin. Struct. Biol.* **20**, 623–631. 10.1016/j.sbi.2010.08.008 PMC3268817 20869868

[bb27] Le Gros, M. A., McDermott, G. & Larabell, C. A. (2005). *Curr. Opin. Struct. Biol.* **15**, 593–600. 10.1016/j.sbi.2005.08.008 16153818

[bb28] Leis, A., Rockel, B., Andrees, L. & Baumeister, W. (2009). *Trends Biochem. Sci.* **34**, 60–70. 10.1016/j.tibs.2008.10.011 19101147

[bb29] Lewis, R. (1997). *Phys. Med. Biol.* **42**, 1213–1243. 10.1088/0031-9155/42/7/001 9253036

[bb30] Li, Y., Tian, Y. & Nie, G. (2012). *Sci. China Life Sci.* **55**, 884–890. 10.1007/s11427-012-4387-7 23108865

[bb31] Liang, X.-J. *et al.* (2010). *Proc. Natl Acad. Sci. USA*, **107**, 7449–7454.

[bb32] Liu, Y., Jiao, F., Qiu, Y., Li, W., Lao, F., Zhou, G., Sun, B., Xing, G., Dong, J., Zhao, Y., Chai, Z. & Chen, C. (2009). *Biomaterials*, **30**, 3934–3945. 10.1016/j.biomaterials.2009.04.001 19403166

[bb33] Liu, Y., Meirer, F., Krest, C. M., Webb, S. & Weckhuysen, B. M. (2016). *Nat. Commun.* **7**, 12634. 10.1038/ncomms12634 PMC5013607 27572475

[bb34] Liu, Y., Nelson, J., Holzner, C., Andrews, J. C. & Pianetta, P. (2013). *J. Phys. D Appl. Phys.* **46**, 494001.

[bb35] Liu, Y., Wang, J., Azuma, M., Mao, W. L. & Yang, W. (2014). *Appl. Phys. Lett.* **104**, 043108. 10.1063/1.4863229 PMC3977758 24753622

[bb36] McDermott, G., Fox, D. M., Epperly, L., Wetzler, M., Barron, A. E., Le Gros, M. A. & Larabell, C. A. (2012). *Bioessays*, **34**, 320–327. 10.1002/bies.201100125 PMC3343367 22290620

[bb37] McIntosh, R., Nicastro, D. & Mastronarde, D. (2005). *Trends Cell Biol.* **15**, 43–51. 10.1016/j.tcb.2004.11.009 15653077

[bb38] Medalia, O., Weber, I., Frangakis, A. S., Nicastro, D., Gerisch, G. & Baumeister, W. (2002). *Science*, **298**, 1209–1213. 10.1126/science.1076184 12424373

[bb39] Meng, J., Liang, X., Chen, X. & Zhao, Y. (2013). *Integr. Biol. (Camb.)*, **5**, 43–47. 10.1039/c2ib20145c PMC3629950 22961501

[bb40] Meng, J., Wang, D.-L., Wang, P. C., Jia, L., Chen, C. & Liang, X.-J. (2010). *J. Nanosci. Nanotechnol.* **10**, 8610–8616. 10.1166/jnn.2010.2691 PMC3042773 21121373

[bb41] Meng, H. *et al.* (2012). *Nanomedicine*, **8**, 136–146. 10.1016/j.nano.2011.08.019 PMC3510664 21930111

[bb42] Meng, H., Xing, G. *et al.* (2010). *ACS Nano*, **4**, 2773–2783. 10.1021/nn100448z 20429577

[bb43] Miao, J., Förster, F. & Levi, O. (2005). *Phys. Rev. B*, **72**, 052103.

[bb44] Nelson, G. J., Harris, W. M., Izzo, J. R., Grew, K. N., Chiu, W. K. S., Chu, Y. S., Yi, J., Andrews, J. C., Liu, Y. & Pianetta, P. (2011). *Appl. Phys. Lett.* **98**, 173109.

[bb45] Ntziachristos, V. (2010). *Nat. Methods*, **7**, 603–614. 10.1038/nmeth.1483 20676081

[bb46] Oberdörster, G., Oberdörster, E. & Oberdörster, J. (2005). *Environ. Health Perspect.* **113**, 823–839. 10.1289/ehp.7339 PMC1257642 16002369

[bb47] Papadimitriou, J. M. & Ashman, R. B. (1989). *Ultrastruct. Pathol.* **13**, 343–372. 10.3109/01913128909048488 2669295

[bb48] Pérez-Berná, A. J., Rodríguez, M. J., Chichón, F. J., Friesland, M. F., Sorrentino, A., Carrascosa, J. L., Pereiro, E. & Gastaminza, P. (2016). *ACS Nano*, **10**, 6597–6611. 10.1021/acsnano.6b01374 27328170

[bb49] Qian, B.-Z. & Pollard, J. W. (2010). *Cell*, **141**, 39–51. 10.1016/j.cell.2010.03.014 PMC4994190 20371344

[bb50] Qiu, J., Deng, B., Yang, Q., Yan, F., Li, A. & Yu, X. (2011). *Nucl. Instrum. Methods Phys. Res. B*, **269**, 2662–2666.

[bb51] Rarback, H., Cinotti, F., Jacobsen, C., Kenney, J. M., Kirz, J. & Rosser, R. (1987). *Biol. Trace Elem. Res.* **13**, 103–113. 10.1007/BF02796625 24254669

[bb52] Ruffell, B., Affara, N. I. & Coussens, L. M. (2012). *Trends Immunol.* **33**, 119–126. 10.1016/j.it.2011.12.001 PMC3294003 22277903

[bb53] Schneider, G., Guttmann, P., Heim, S., Rehbein, S., Mueller, F., Nagashima, K., Heymann, J. B., Müller, W. G. & McNally, J. G. (2010). *Nat. Methods*, **7**, 985–987. 10.1038/nmeth.1533 PMC7337972 21076419

[bb54] Scott, M. C., Chen, C.-C., Mecklenburg, M., Zhu, C., Xu, R., Ercius, P., Dahmen, U., Regan, B. C. & Miao, J. (2012). *Nature (London)*, **483**, 444–447. 10.1038/nature10934 22437612

[bb55] Smith, E. A., McDermott, G., Do, M., Leung, K., Panning, B., Le Gros, M. A. & Larabell, C. A. (2014). *Biophys. J.* **107**, 1988–1996. 10.1016/j.bpj.2014.09.011 PMC4213715 25418180

[bb56] Stephens, D. J. & Allan, V. J. (2003). *Science*, **300**, 82–86. 10.1126/science.1082160 12677057

[bb57] Uchida, M., McDermott, G., Wetzler, M., Le Gros, M. A., Myllys, M., Knoechel, C., Barron, A. E. & Larabell, C. A. (2009). *Proc. Natl Acad. Sci. USA*, **106**, 19375–19380. 10.1073/pnas.0906145106 PMC2780763 19880740

[bb58] Varsano, N., Dadosh, T., Kapishnikov, S., Pereiro, E., Shimoni, E., Jin, X., Kruth, H. S., Leiserowitz, L. & Addadi, L. (2016). *J. Am. Chem. Soc.* **138**, 14931–14940. 10.1021/jacs.6b07584 PMC6530486 27934213

[bb59] Wang, J. *et al.* (2006). *Biochem. Pharmacol.* **71**, 872–881. 10.1016/j.bcp.2005.12.001 16436273

[bb60] Wang, L., Zhang, T., Li, P., Huang, W., Tang, J., Wang, P., Liu, J., Yuan, Q., Bai, R., Li, B., Zhang, K., Zhao, Y. & Chen, C. (2015). *ACS Nano*, **9**, 6532–6547. 10.1021/acsnano.5b02483 25994391

[bb61] Weissleder, R., Nahrendorf, M. & Pittet, M. J. (2014). *Nat. Mater.* **13**, 125–138. 10.1038/nmat3780 24452356

[bb62] Yao, S. K., Fan, J. D., Zong, Y. B., He, Y., Zhou, G. Z., Sun, Z. B., Zhang, J. H., Huang, Q. J., Xiao, T. Q. & Jiang, H. D. (2016). *Appl. Phys. Lett.* **108**, 123702.

[bb63] Yin, J.-J., Lao, F., Fu, P. P., Wamer, W. G., Zhao, Y., Wang, P. C., Qiu, Y., Sun, B., Xing, G., Dong, J., Liang, X.-J. & Chen, C. (2009). *Biomaterials*, **30**, 611–621. 10.1016/j.biomaterials.2008.09.061 PMC8785860 18986699

[bb64] Yin, J.-J., Lao, F., Meng, J., Fu, P. P., Zhao, Y., Xing, G., Gao, X., Sun, B., Wang, P. C., Chen, C. & Liang, X.-J. (2008). *Mol. Pharmacol.* **74**, 1132–1140. 10.1124/mol.108.048348 PMC12696667 18635669

[bb65] Zhang, W., Sun, B., Zhang, L., Zhao, B., Nie, G. & Zhao, Y. (2011). *Nanoscale*, **3**, 2636–2641. 10.1039/c1nr10239g 21541378

[bb67] Zhang, L.-J., Xu, Z.-J., Zhang, X.-Z., Yu, H.-N., Zou, Y., Guo, Z., Zhen, X.-J., Cao, J.-F., Meng, X.-Y., Li, J.-Q., Chen, Z.-H., Wang, Y. & Tai, R.-Z. (2015). *Nucl. Sci. Tech.* **26**, 040101.

[bb66] Zhang, X. Z., Xu, Z. J., Tai, R. Z., Zhen, X. J., Wang, Y., Guo, Z., Yan, R., Chang, R., Wang, B., Li, M., Zhao, J. & Gao, F. (2010). *J. Synchrotron Rad.* **17**, 804–809. 10.1107/S0909049510031250 20975228

[bb68] Zhao, Y., Brun, E., Coan, P., Huang, Z., Sztrókay, A., Diemoz, P. C., Liebhardt, S., Mittone, A., Gasilov, S., Miao, J. & Bravin, A. (2012). *Proc. Natl Acad. Sci. USA*, **109**, 18290–18294. 10.1073/pnas.1204460109 PMC3494902 23091003

[bb69] Zhao, F.-J., Moore, K. L., Lombi, E. & Zhu, Y.-G. (2014). *Trends Plant Sci.* **19**, 183–192. 10.1016/j.tplants.2013.12.001 24394523

