# Supplementary material for: Three-dimensional ultrastructural imaging reveals the nanoscale architecture of mammalian cells
Source: IUCrJ. 2018 Jan 10;5(Pt 2):141–9. doi: 10.1107/S2052252517017912 (PMC5947718; doi:10.1107/S2052252517017912)
Supplement: Supplementary file 1 [file m-05-00141-sup1.pdf]

IUCrJ

Volume 5 (2018)

Supporting information for article:

Three-dimensional ultrastructural imaging reveals nanoscale architecture of mammalian cells

Shengkun Yao, Jiadong Fan, Zhiyun Chen, Yunbing Zong, Jianhua Zhang, Zhibin Sun, Lijuan Zhang, Renzhong Tai, Zhi Liu, Chunying Chen and Huaidong Jiang

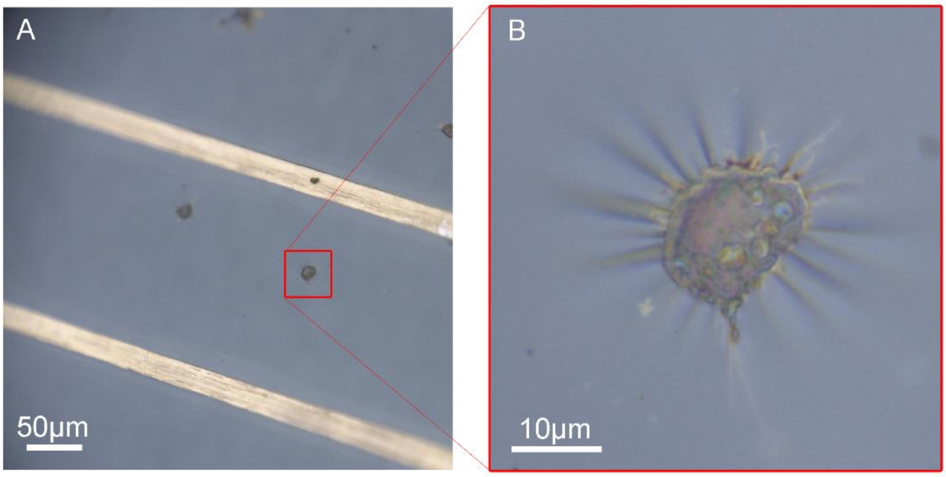

Figure S1 Optical images of the selected macrophage.

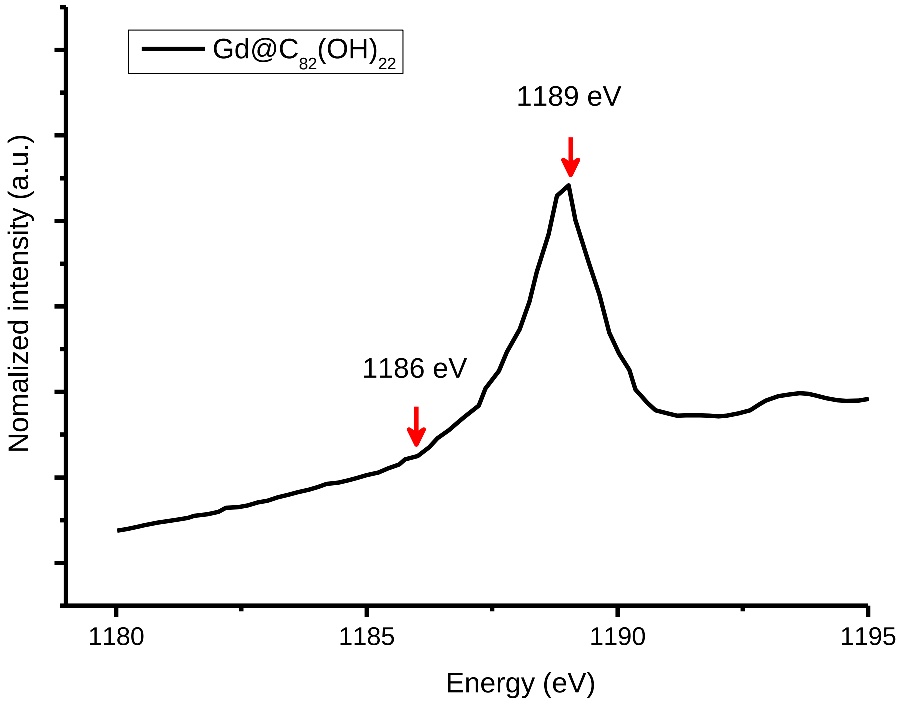

Figure S2 . The energies in the experiment—1186 eV and 1189 eV—were determined by the M_2 absorption edge of Gd based on the TEY signals of Gd@$C_{82}(OH)_{22}$ powder.

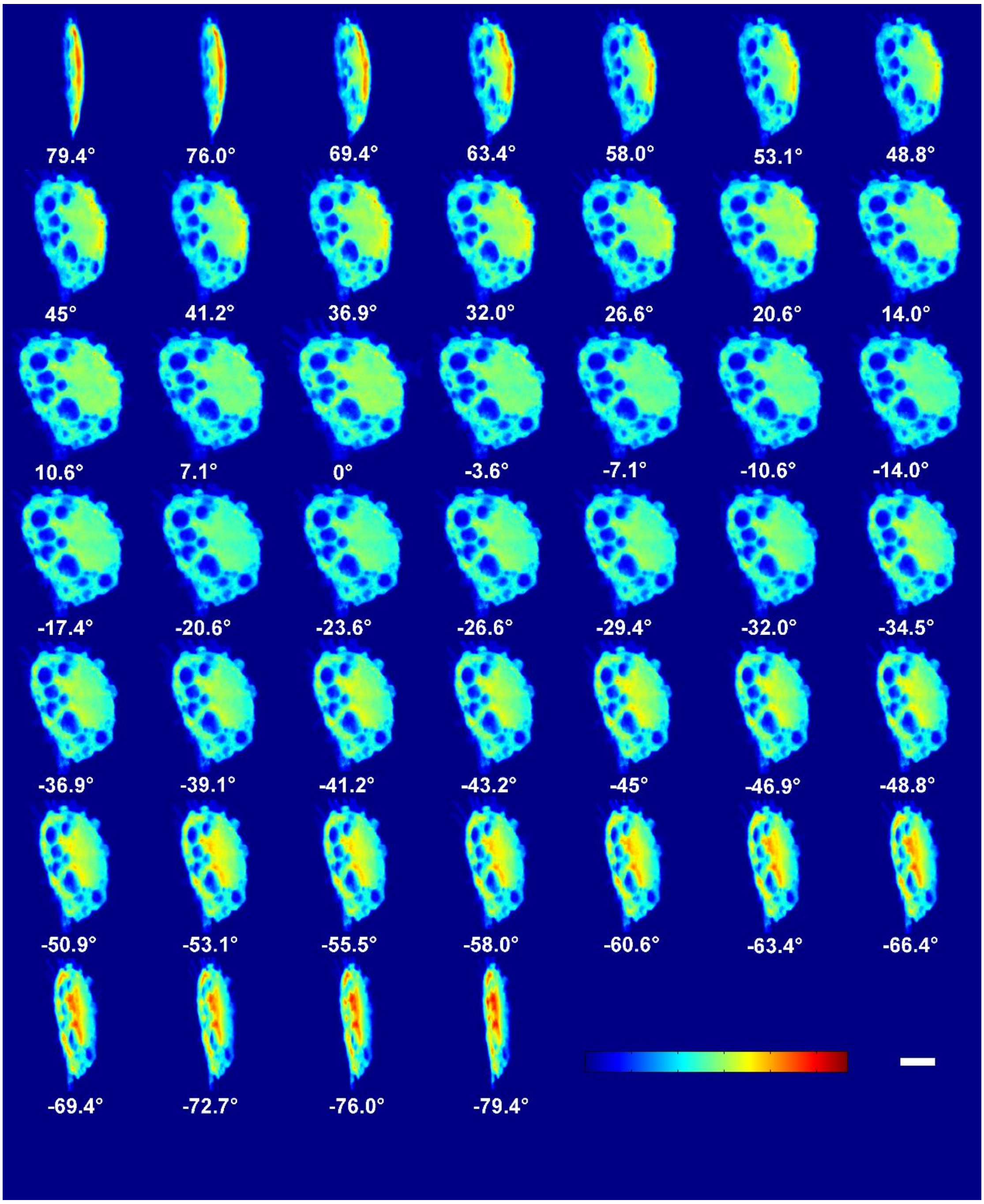

Figure S3 Forty-six projections of the macrophage for tomographic reconstruction at 1186 eV. The projections range from -79.4° to +79.4° and are separated by equally sloped increments (scale bar, 5.0 μm).

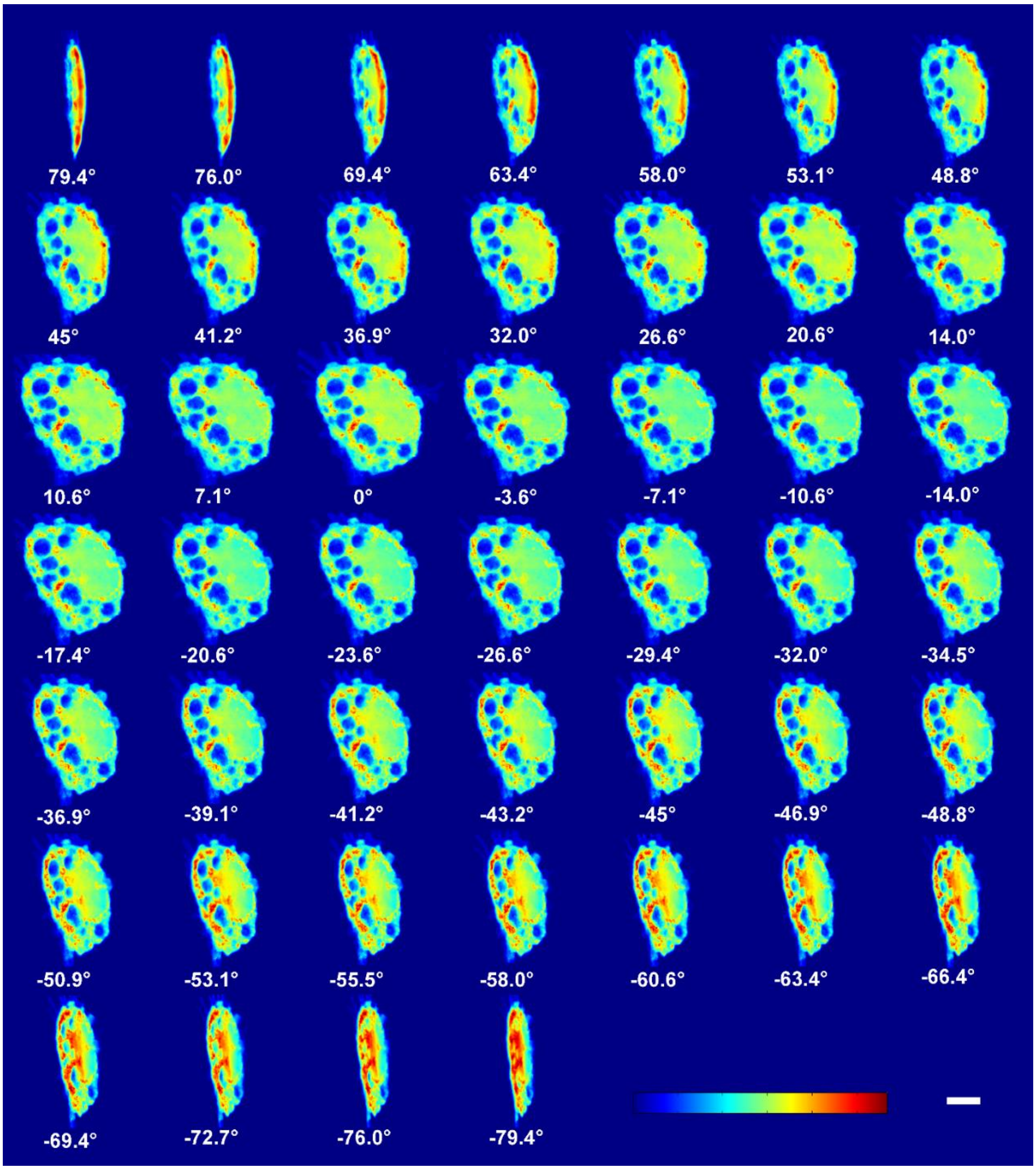

Figure S4 Forty-six projections of the macrophage for tomographic reconstruction at 1189 eV. The projections range from -79.4° to +79.4° and are separated by equally sloped increments (scale bar, 5.0 μm).

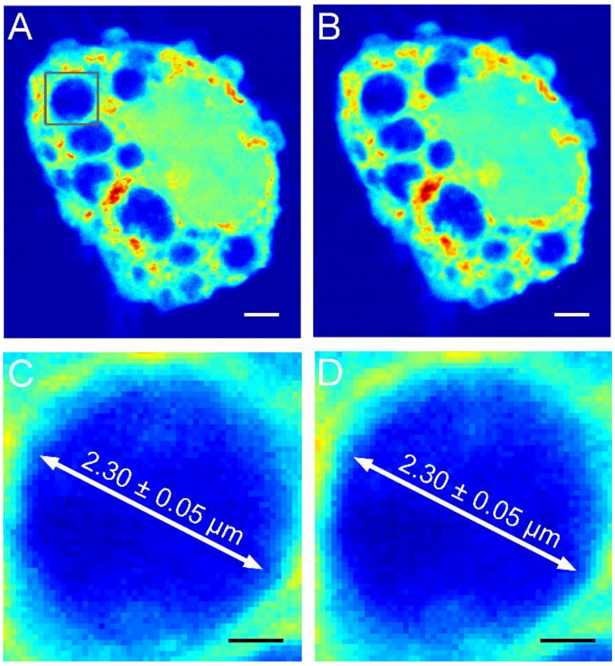

Figure S5 Estimation of the radiation effects during the experiment. Two 0° projections were taken before (A) and after (B) the acquisition of full datasets to measure the effect of radiation on the macrophage. The overall shapes were in good agreement, indicating there no structural changes could be distinguished at this resolution. The zoomed-in regions (C, D) with low density were highly consistent, confirming that no detectable radiation damage occurred in the cell (scale bar for A and B, 2.0 µm; for C and D, 0.5 µm).

To examine the quality of the EST reconstruction, projections were calculated from the 3D images at the same experimental angles. A factor, Err, was defined to show the divergence of the experimental and calculated projections by

,

where μ_1 and μ_2 represent the experimental and calculated projections, respectively. The average Err for all tilt angles was ~5%, and the Err of all projections at two energies was distributed around 4%–6% (Fig. S6C). The slight discrepancy revealed by Err confirmed the accuracy of the tomographic reconstruction. Representative experimental and calculated projections at a tilt angle of -14.04° at 1186 eV are shown in Figs. S6A and S6B. No obvious differences were observed at this resolution. The consistency between the experimental and calculated projections confirmed the high quality of the EST reconstruction.

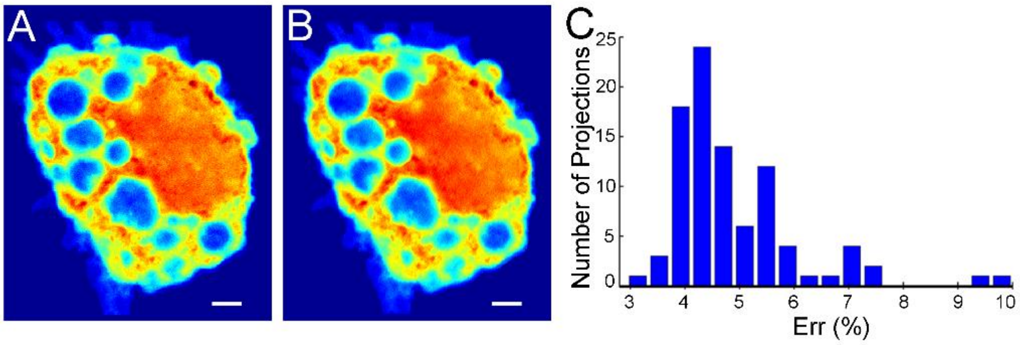

Figure S6 Measurement of the quality of the tomographic reconstruction. Representative experimental (A) and calculated (B) projections at an angle of -14.04°. A function (Err) was defined to show divergence of the experimental and calculated projections. (C) The Err histogram. The experimental and calculated projections were in good agreement, indicating the high validity of the tomographic reconstruction. The Err values were in the range of ~4%–6%, further confirming the high quality of the EST reconstruction (scale bar, 2.0 μm).

To quantify the resolution of the 3D images, a primary lysosome was scanned along the X, Y and Z axes, where Y is the tilt axis, and Z is the direction of the beam (Fig. S7). Along the X and Y axes, a resolution of ~75 nm was determined, and along the Z axis, the resolution was approximately 80 nm, based on the 10%–90% criteria (Figs. S7B, S7C and S7D). To further quantify the resolution, the power spectrum density (PSD) of one slice in the XY plane (i.e., perpendicular to the beam direction) at 1189 eV was calculated, revealing that the resolution was approximately 76 nm (Fig. S8). In summary, a resolution of ~75–80 nm in the 3D structure was obtained, and the missing wedge problem was alleviated by EST iterations. The morphologies and subcellular structures of the macrophage could be analyzed both quantitatively and qualitatively at this resolution.

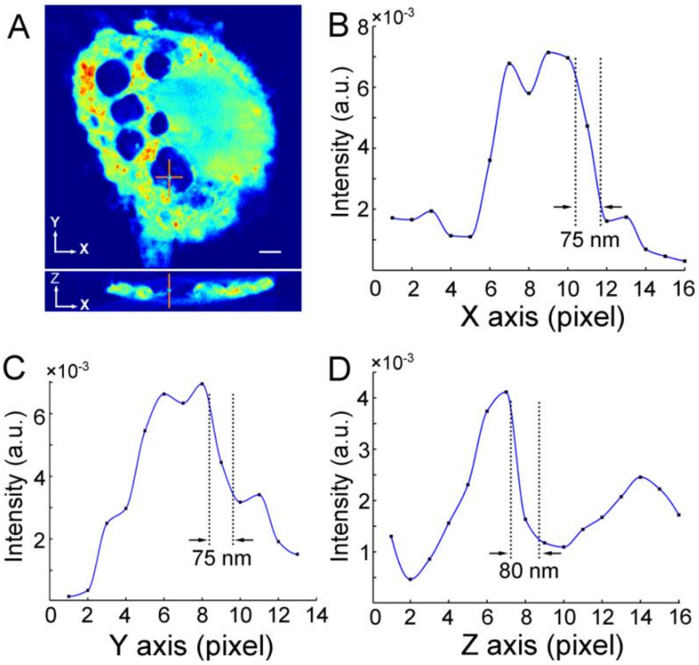

Figure S7 The resolution of the 3D tomographic images. (A) A primary lysosome was scanned along three axes to measure the 3D resolution using the 10%–90% density change criteria. (B and C) A resolution of ~75 nm was obtained along the X and Y axes. (D) A resolution of ~80 nm was obtained along the Z axis. The lower resolution along the Z axis was attributed to missing projections in that direction (scale bar, 2.0 μm).

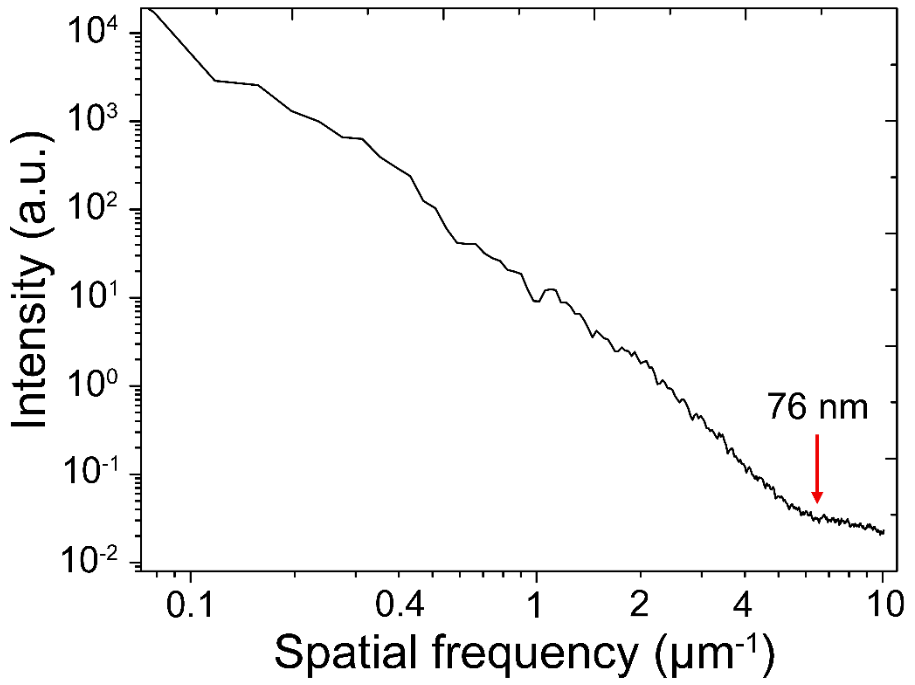

Figure S8 Quantification of the resolution by PSD. The PSD of a slice perpendicular to beam direction at 1189 eV was calculated and indicated that a resolution of ~76 nm was obtained.

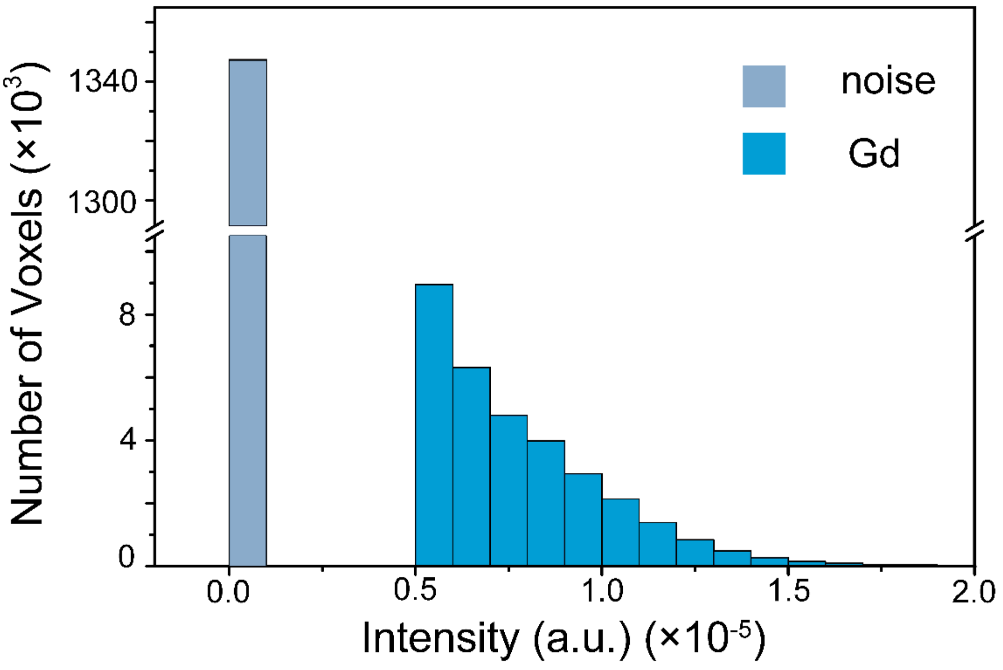

Figure S9 The method used to distinguish the pixels that contained Gd. The intensity histogram was plotted to determine the threshold. The pixels considered to contain no Gd, whose intensity was less than the threshold, were set to zero. In this case, $0.5{\times}10^{-5}$ was set as the threshold.

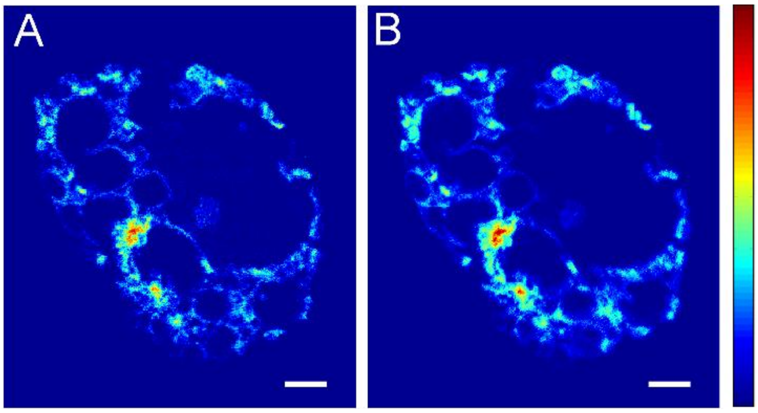

Figure S10 Comparison of the 2D distribution of Gd (A) with that of a 2D projection from the calculated 3D Gd distribution (B). The consistency indicates the appropriateness of this method (scale bar, 2.0 μm).

Table S1 $Gd@C_{82}(OH)_{22}$ can activate primary mouse macrophages to produce significant numbers of pro-inflammatory cytokines.

Cytokines	$Gd@C_{82}(OH)_{22}$
GM-CSF	30.8
G-CSF	154.8
IL-1β	16.2
IL-10	6.2
MCP-1	99.6
RANTES	11.5
IL-1α	29.6
IL-6	3.6
MIP-1α	87.3
TNF-α	449.1
TNF-R II	5.3
